# When Problem Size Matters: Differential Effects of Brain Stimulation on Arithmetic Problem Solving and Neural Oscillations

**DOI:** 10.1371/journal.pone.0120665

**Published:** 2015-03-19

**Authors:** Bruno Rütsche, Tobias U. Hauser, Lutz Jäncke, Roland H. Grabner

**Affiliations:** 1 Research on Learning and Instruction, Institute for Behavioral Sciences, ETH Zurich, Zurich, Switzerland; 2 Neuroscience Center Zurich (ZNZ), University of Zurich and ETH Zurich, Zurich, Switzerland; 3 Wellcome Trust Centre for Neuroimaging, Institute of Neurology, University College London, London, United Kingdom; 4 University Clinics for Child and Adolescent Psychiatry (UCCAP), University of Zurich, Zurich, Switzerland; 5 Division Neuropsychology, Institute of Psychology, University of Zurich, Zurich, Switzerland; 6 International Normal Aging and Plasticity Imaging Center (INAPIC), University of Zurich, Zurich, Switzerland; 7 Center for Integrative Human Physiology (ZIHP), University of Zurich, Zurich, Switzerland; 8 University Research Priority Program (URPP), Dynamic of Healthy Aging, University of Zurich, Zurich, Switzerland; 9 Department of Special Education, King Abdulaziz University, Jeddah, Saudi Arabia; 10 Section of Educational Neuroscience, Department of Psychology, University of Graz, Graz, Austria; 11 Department of Educational Psychology, Institute of Psychology, Georg-August-University of Göttingen, Göttingen, Germany; University of British Columbia, CANADA

## Abstract

The problem size effect is a well-established finding in arithmetic problem solving and is characterized by worse performance in problems with larger compared to smaller operand size. Solving small and large arithmetic problems has also been shown to involve different cognitive processes and distinct electroencephalography (EEG) oscillations over the left posterior parietal cortex (LPPC). In this study, we aimed to provide further evidence for these dissociations by using transcranial direct current stimulation (tDCS). Participants underwent anodal (30min, 1.5 mA, LPPC) and sham tDCS. After the stimulation, we recorded their neural activity using EEG while the participants solved small and large arithmetic problems. We found that the tDCS effects on performance and oscillatory activity critically depended on the problem size. While anodal tDCS improved response latencies in large arithmetic problems, it decreased solution rates in small arithmetic problems. Likewise, the lower-alpha desynchronization in large problems increased, whereas the theta synchronization in small problems decreased. These findings reveal that the LPPC is differentially involved in solving small and large arithmetic problems and demonstrate that the effects of brain stimulation strikingly differ depending on the involved neuro-cognitive processes.

## Introduction

The development of mathematical competencies is a major goal of formal schooling and an important prerequisite for success in various areas of life [1]. An essential step in this development lies in the acquisition of arithmetic skills which has been in the focus of cognitive and educational research for several decades (for a review see e.g. [2]). This research has produced much evidence showing that arithmetic problem solving relies on different (neuro-)cognitive processes depending on the type of arithmetic problem.

The presumably most robust and well-established finding in this domain is the problem size effect (PSE) [3,4]. It is reflected in longer response latencies and lower accuracies when solving arithmetic problems with larger operands (i.e. sums > 10; large problems) compared to problems with smaller operands (i.e. sums ≤ 10; small problems). To date, several explanations and models on the cause of this effect have been put forward (see e.g. [5–9]). The application of different strategies in problems of different size is assumed to be a major contributing factor to the PSE [10,11]. In particular, it has been shown that small arithmetic problems are primarily solved by fast and efficient direct retrieval of the answers from long-term memory, whereas larger problems are more often solved through time-consuming and error-prone non-retrieval procedures such as counting and transformation (e.g. decomposition of an arithmetic problem using known facts: 18 + 13 = 18 + 2 + 11).

Functional neuroimaging studies have revealed that the PSE is associated with specific neurophysiological activation patterns in the left posterior parietal cortex (LPPC). Specifically, there is a large body of evidence that left-hemispheric perisylvian language regions such as the supramarginal and angular gyrus were mainly activated in small arithmetic problems [12–14], whereas the intraparietal sulcus showed greater activation in large problems [12,13,15,16]. Neural activation of the former areas have generally been assumed to reflect the retrieval of arithmetic facts from long-term memory, while activation of the latter region was related to quantity processing and manipulations during procedural calculation [17–19].

Different problem sizes are known to be accompanied by dissociable oscillatory patterns in the electroencephalogram (EEG) [20–22]. For example, Grabner and De Smedt [21] performed a study that was specifically designed to investigate the oscillatory correlates of problem size and strategy use. They presented small and large addition and subtraction problems and gathered trial-by-trial verbal strategy reports. The authors used event-related (de-)synchronization (ERS/ERD) measures, which reflect the extent to which local synchrony in a specific frequency band is gained (ERS) or lost (ERD) from a pre-stimulus reference interval to an activation interval during task processing. Using this approach, Grabner and De Smedt [21] found a clear dissociation of problem size or strategy and EEG frequency band: small problems were characterized by an increased left-hemispheric theta ERS (3–6 Hz) and large problems were accompanied by a widespread lower-alpha ERD (8–10 Hz). Interestingly, the same neural effects hold true for retrieval problems (increased theta ERS) and procedurally solved problems (lower-alpha ERD) (for similar results see also [20]). The finding of greater theta ERS at parietal areas for small/retrieval problems was interpreted to reflect the retrieval of arithmetic facts mediated by task-relevant areas such as the LPPC. This interpretation corresponds to findings associating theta ERS with memory performance in general [23–27] and with the retrieval of lexical-semantic information from long-term memory in particular [25,26,28,29]. The stronger lower-alpha desynchronization in large/procedural problems was seen as the consequence of the more effortful nature of procedural calculation. This is in line with the common interpretation of lower-alpha ERD as marker of basic attentional processes, with greater ERD values indicating increased brain activation and attentional involvement [30–34].

Taken together, distinct patterns of neural activation have been shown to emerge in the LPPC depending on the problem size and the associated cognitive processes. A central restriction of the aforementioned studies using neuroimaging methods lies in their correlational nature (see e.g. [35]). Accordingly, it is unclear whether the previously observed activation patterns in the LPPC are causally related to arithmetic performance. Non-invasive brain stimulation techniques, however, can be used to investigate the causative role of cortical areas for specific cognitive functions. In recent years, especially transcranial direct current stimulation (tDCS) has gained increasing popularity [36–39]. At a neurophysiological level, anodal tDCS has been shown to increase the excitability of the neuronal tissue located beneath the stimulation location [38,40,41]. At a cognitive level, anodal stimulation has typically been found to improve a wide variety of cognitive functions such as working memory [42–44], language learning [45,46] or attention [47,48] as well as clinical disorders such as depression [49] or aphasia [50]. Recently, however, there is a growing number of tDCS studies showing that anodal tDCS does not only have beneficial effects, but can also impair cognitive performance in certain tasks [51–55].

In the mathematical domain, there have been high expectations that this method could in future be used to support the treatment of learning disorders such as developmental dyscalculia, which is characterized by deficits in both basic number processing and arithmetic skills [36,56]. Indeed, recent studies have provided first evidence of positive effects of tDCS on basic number processing as well as arithmetic performance [51,57,58]. Most relevant to the current study, Hauser et al. [58] have shown that anodal tDCS over the LPPC improved solution times in large (procedural) subtraction problems. The effects of parietal tDCS on small (retrieval) problems, however, have not been investigated so far. Furthermore, despite the popularity of these techniques, relatively little is known about how the induced behavioral changes relate to modulations in neural activity. However, there are increasing endeavors to investigate the neurophysiologic effects of brain stimulation techniques by combining them with neuroimaging methods such as EEG [35,44,59–61].

Against this background, the aim of the present study was to provide further evidence for the neuro-cognitive dissociation in the LPPC between small and large arithmetic problems and, thus, to further clarify the role of the LPPC in arithmetic problem solving. To this end, participants underwent anodal and sham tDCS over the LPPC. Afterwards, neural activity was recorded using EEG while the participants solved small and large arithmetic problems. We hypothesized that anodal tDCS (compared to sham tDCS) changes oscillatory EEG activity in the theta band while solving small problems and in the lower-alpha band while solving large problems. These modulations are expected to be accompanied by performance improvements in both small and large arithmetic problems.

## Method

### Participants

Twenty-six healthy participants without any neurologic, psychiatric or mathematical (learning) disorders were recruited among students of the University of Zurich. None of the participants were taking any medication affecting the central nervous system at the time of participation. All participants were consistent right-handers as determined by the Annett Handedness Inventory [62]. One participant was excluded due to health issues unrelated to the experiment in one session and two male subjects were excluded due to excessive movement- and muscle-related EEG-artifacts from the overall analysis. The final sample was composed of 17 female and 6 male participants (age: M = 21.78y, SD = 2.66, range = 19–32). The study was approved by the ethics commission of the ETH Zurich, Switzerland (EK 2011-N-52). All subjects were thoroughly informed about the study, gave written informed consent and received course credit as compensation for their participation.

### Procedure

**Fig 1:**
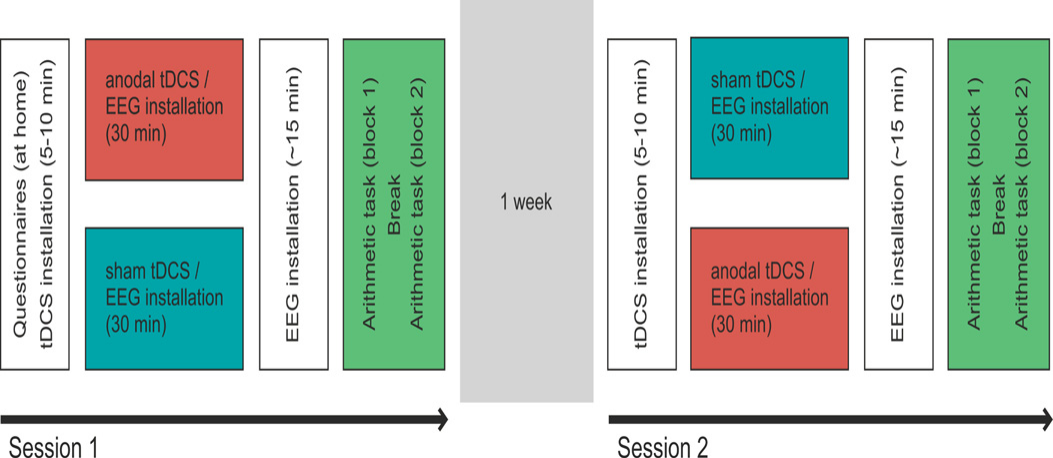
Schematic depiction of the experimental procedure. The study consisted of two sessions one week apart. Participants underwent one session of anodal and sham tDCS each (order counterbalanced). The arithmetic task was presented after stimulation.

The within-subjects experimental procedure is depicted in Fig. 1. The study consisted of two 2-hour sessions with an intersession interval of one week to minimize training effects. Participants underwent one session of anodal and sham tDCS each. Both conditions were counterbalanced across subjects to control for order effects. In both sessions, tDCS electrodes were fastened under an EEG cap and the subjects were seated in an electromagnetically shielded room. During the DC stimulation, all EEG electrodes not directly located over the tDCS electrodes were inserted into the EEG cap and participants had the chance to familiarize themselves with the experimental task by means of six practice trials. The practice trials involved 6 arithmetic problems with the same operand (i.e., 2+2 or 15+15). Upon termination of the DC stimulation, the remaining EEG electrodes were mounted (duration about 15 min). The experimental task and EEG recording was started directly afterwards (i.e., about 45 minutes after the beginning of DC stimulation). Thus, both the behavioral task and the EEG measurement were performed “offline”, i.e., after the DC stimulation.

In the experimental task, the participants were presented with addition and subtraction problems of small and large problem size. Both operations were presented in two separate blocks to avoid operation switching effects. The blocks had a length of about 7 minutes and were separated by a short break. The sequence of the operation blocks over both sessions was counterbalanced across participants (i.e., either addition / subtraction or subtraction / addition in both sessions). Small and large problems within a block were intermixed and presented in a fixed pseudorandomized order to prevent that problems with the same result are presented consecutively.

**Fig 2:**
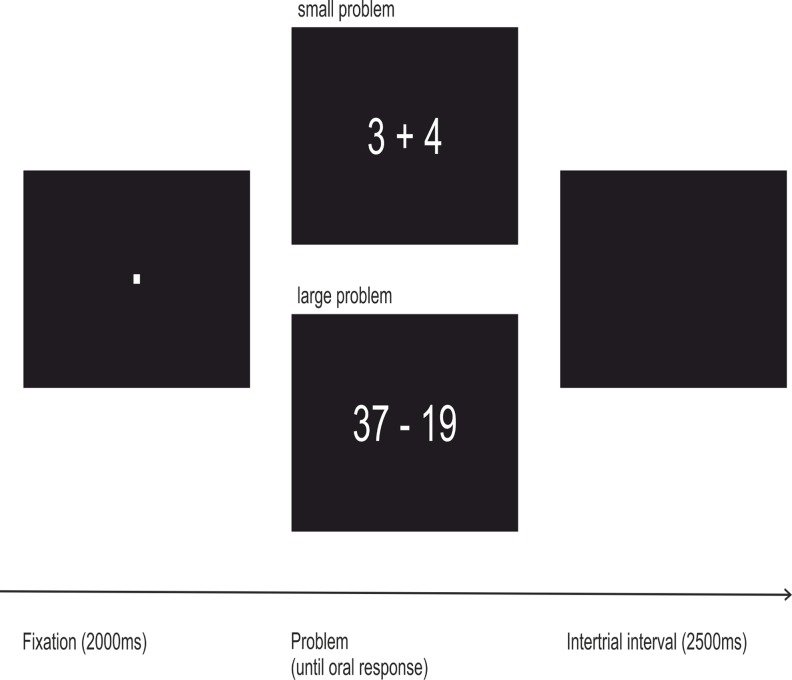
Schematic depiction of a trial of the arithmetic task used in the present study. 72 small (sums ≤ 10) and 72 large problems (sums > 10, with carry) were presented in a fixed pseudorandomized order.

Participants were instructed to solve the problems as accurately and fast as possible and to speak the answer into a microphone as soon as they had found the solution. A single trial (see Fig. 2) consisted of the presentation of a fixation point for 2000 ms, followed by the arithmetic problem that disappeared as soon as an oral response was registered. The problem was automatically faded out if no response was recorded within 10000 ms. Subsequently, an inter-trial interval with a length of 2500 ms was presented. E-Prime 2.0 software (Psychology Software Tools, Pittsburgh, PA) was used for stimulus delivery. Response latencies represented the time from problem onset to voice onset as registered by a voice key. Throughout the task, the experimenter recorded the correctness of each oral response and additionally noted rare problems arising with the collection of the vocal latencies (e.g. too late registration). The EEG cap was removed upon completion of the arithmetic task. At the end of the experiment, all participants were debriefed and received course credit.

### Stimulus material

In each session, participants solved 72 small and 72 large problems, composed of 36 addition and 36 subtraction problems each. The subtraction problems were created by mirroring the addition problems (e.g. 3 + 5 = 8 to 8–5 = 3).

Small problems were defined as one-digit/one-digit problems with addends between 2 and 8. Problems with 0 or 1 as operands and tie problems were excluded. These small problems have been reported to be predominantly solved by means of memory retrieval (about 90%; [10,21,63]). Since a maximum of 24 small problems can be generated under these constraints, 12 problems were randomly chosen and repeated once in one session, whereas the remaining 12 problems were used in the other session.

For the large problems, 36 problems from a pool of every possible two-digit/two-digit carry problem with addends ranging from 12 to 29 were randomly selected [10]. Only one of each commutative pair was chosen (e.g. 12 + 19 or 19 + 12). This definition of large problems has been shown to result in a large proportion of self-reported procedure use (about 76%; [21]). Notably, the administered large problems are comparable in difficulty to the problems used by a recent tDCS study on procedural calculation [58].

### Transcranial direct current stimulation

A battery-driven electrical DC stimulator (NeuroConn GmbH, Ilmenau, Germany) was used to modulate cortical excitability of the LPPC. The direct current was applied through a pair of conductive rubber electrodes placed in saline-soaked sponges. Both electrodes were fastened under an EEG cap and were, if necessary, further fixated using elastic rubber bands. Anodal tDCS was applied for 30 min at 1.5 mA intensity, while sham tDCS was applied for 30 s at the same intensity, similarly to the procedure reported by Hauser et al. (2013). In both conditions, additional fade-in or fade-out periods of 10 s were employed during which current intensity was linearly in- or decreased. Using this procedure, active and sham stimulation are known not to be distinguishable [64], because most subjects experience the common tingling sensation only at the beginning of the DC stimulation due to a rapid habituation to the sensation [38,65]. In the present study, we asked the participants at the end of both sessions to rate the strength of the sensation induced by the stimulation on a five-point Likert scale. The subjects were not able to consciously distinguish anodal from sham stimulation (Wilcoxon signed rank test: *V* = 77.5, *p* > 0.1).

The active electrode (7x5 cm) was centered over positions P5 and CP5 of the extended 10–20 system for scalp electrodes. Several studies using different methodologies have shown that the cortical projections of these locations lie over the LPPC [66–68]. The reference electrode (10x10 cm) was placed over the right supraorbital area, which can be considered as a standard reference area [38,39]. The combination “parietal lobe—contralateral supraorbital area” has been successfully used in other studies (see e.g. [58,69]).

### EEG recording and preprocessing

Continuous EEG was recorded using a BioSemi ActiveTwo system (BioSemi, Amsterdam, The Netherlands) with 64 active electrodes mounted in elastic Biosemi headcaps with electrode positions according to the extended 10–20 system. Three additional active electrodes were used to record vertical and horizontal electrooculograms (EOG). Two electrodes were placed horizontally at the outer canthi of both eyes and one was placed above the nasion between the inner canthi of both eyes. In BioSemi systems, instead of a recording reference electrode, a feedback loop consisting of the Common Mode Sense active electrode and the Driven Right Leg passive electrode is used. EEG and EOG signals were sampled at 256 Hz.

EEG data were preprocessed in EEGLAB 10.2.5.7b [70]. A high-pass finite impulse response (FIR) filter with a lower edge of 0.5 Hz (transition bandwidth: 0.2 Hz, order: 6.0) and a notch FIR filter between 45 and 55 Hz (transition bandwidth: 1 Hz, order: 12.0) was applied to eliminate direct current shifts and power-line noise. Gross artifacts and bad channels in continuous data were rejected by visual inspection to achieve a clean independent component analysis. On the pruned data, ICA was performed and the EEGLAB plugin ADJUST was used to assist in identifying and rejecting independent components reflecting stereotypical artifacts such as eye blinks and eye movements [71]. The data were re-referenced to average reference and exported to the software g.BSanalyze 3.10.00 (g.tec medical engineering GmbH, Schiedlberg, Austria). Within g.BSanalyze, trials of 10000 ms length (3000 ms before and 7000 ms after problem onset) were extracted from the continuous data. All trials were again visually inspected for remaining artifacts. Trials with large artifacts or with less than 500 ms of artifact-free data within the reference or activation interval (see below) were discarded (about 18% of the trials).

ERS/ERD values for the theta (4–7 Hz) and lower-alpha (8–10 Hz) frequency bands were calculated, since these frequency bands have been shown to be particularly sensitive to arithmetic problem solving and strategy use [20–22]. For each epoch, power values in these frequency bands were obtained by digitally bandpass filtering using a Fast Fourier Transformation (FFT), then squaring and averaging over consecutive data points according to a backwards-oriented moving window of 500 ms length. The reference interval (R) was defined as the time period from 2000 ms to 500 ms before problem onset, whereas the activation interval (A) was defined as the interval from problem onset up to 125 ms before the oral response. The last 125 ms were discarded to diminish motor- and speech-related artifacts possibly affecting the activation interval. Therefore, the activation interval differed in length between individuals and trials, which has the advantage—in contrast to a fixed activation interval—that all and only task-specific cognitive processes are included in the analysis (for a similar procedure see [20,21]). Afterwards, the median value of all power values within the reference or activation interval was calculated for each trial (horizontal averaging). These values were then averaged over all trials, separately for both stimulation conditions (anodal, sham) and problem sizes (small, large; vertical averaging). ERS/ERD was calculated as %ERS/ERD = (A-R)/R x 100. ERS/ERD values at channels CP5 and P5, which directly underlay the active tDCS electrode, were averaged to form the region of interest.

### Statistical analysis

All further statistical analyses were carried out in statistics software R 2.15.2 [72]. For the analysis of response latencies, only correctly solved trials were used. Moreover, trials with response latencies faster or slower than 3 standard deviations from each subject’s mean (separately for each problem size; 2.02% of the residual trials) and trials marked by the experimenter as unreliable (0.86% of the residual trials) were discarded (cf. [73]). Analysis of the PSE were conducted on data collapsed across stimulation conditions. Due to the short-lasting effects of tDCS on EEG (see [44]), we conducted our EEG analysis only on the first block of each session.

## Results

### Behavioral measures

Confirming the well-established PSE, we found that large problems took longer to solve (M = 2899 ms, SD = 751) than small problems (M = 997 ms, SD = 119; *t*(22) = 13.2, *p* < 0.01, Cohen’s *d* = 2.76). Furthermore, solving large problems (M = 88.32%, SD = 5.26) was characterized by lower solution rates than solving small problems (M = 98.31%, SD = 0.94; *t*(22) = 9.26, *p* < 0.01, *d* = 1.93).

**Fig 3:**
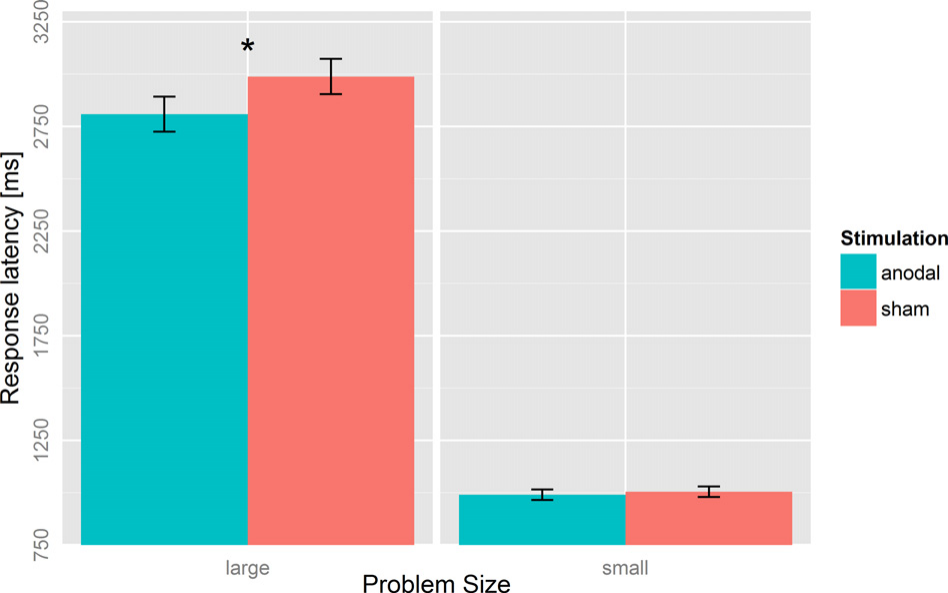
Mean response latency after anodal and sham stimulation for small and large problems. Response latency in large problems was decreased after anodal compared to sham stimulation. Error bars indicate standard errors (SE). *p < 0.05, **p < 0.01.

**Fig 4:**
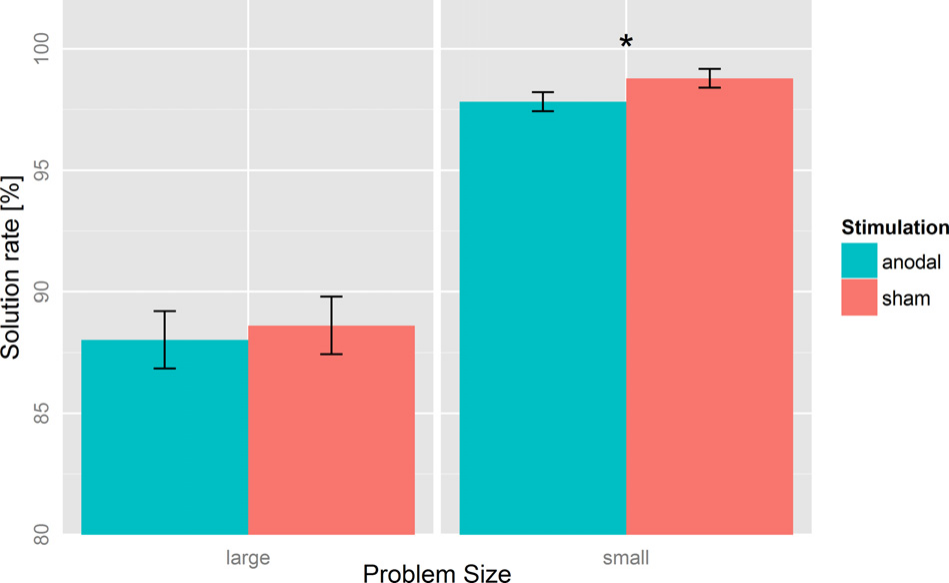
Mean solution rate after anodal and stimulation for small and large problems. Solution rate in small problems was decreased after anodal compared to sham stimulation. Error bars indicate standard errors (SE). *p < 0.05, **p < 0.01.

In large problems, we found an improvement in response latencies after anodal (M = 2809 ms, SD = 707) compared to sham stimulation (M = 2989 ms, SD = 842; *t*(22) = 2.14, *p* = 0.04, *d* = *0*.45; Fig. 3 left panel). Solution rates in large problems remained unaffected by stimulation (*t*(22) = 0.50, *p* = 0.62, *d = 0*.*10*; Fig. 4 left panel).

In small problems, the response latencies after anodal (M = 990 ms, SD = 135) and sham stimulation (M = 1004 ms, SD = 132; *t*(22) = 0.56, *p* = 0.58, *d* = *0*.*1*2; Fig. 3 right panel) did not differ. However, solution rates were significantly reduced after anodal (M = 97.82%, SD = 1.31) compared to sham stimulation (M = 98.78%, SD = 1.35; *t*(22) = 2.44, *p* = 0.02, *d* = *0*.*51*; Fig. 4 right panel).

Thus, anodal stimulation enhanced performance in large problems (in terms of response latencies) but decreased performance in small problems (in terms of solution rates).

### Event-related (de-)synchronization (ERS/ERD)

In line with previous EEG studies investigating the PSE, large problems (M = -14.00, SD = 16.64) were accompanied by greater lower-alpha ERD than small problems (M = 3.11, SD = 24.69; *t*(22) = 4.1, *p* < 0.01, *d* = *0*.*86*). In contrast, small problems (M = 24.24, SD = 20.14) were characterized by significantly greater theta ERS than large problems (M = 9.60, SD = 14.23; *t*(22) = 3.23, *p* < 0.01, *d* = *0*.*67*).

**Fig 5:**
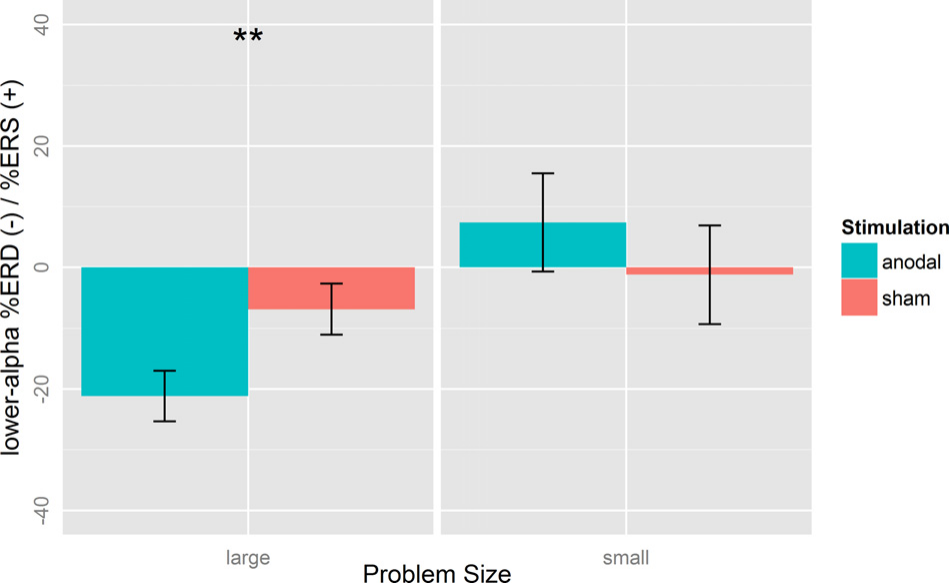
Mean %Event-related (de-)synchronization (%ERS/ERD) of the lower-alpha band (8–10 Hz) after anodal and sham stimulation for small and large problems at electrodes CP5/P5. Lower-alpha ERD in large problems was increased after anodal compared to sham stimulation. Error bars indicate standard errors (SE). *p < 0.05, **p < 0.01.

**Fig 6:**
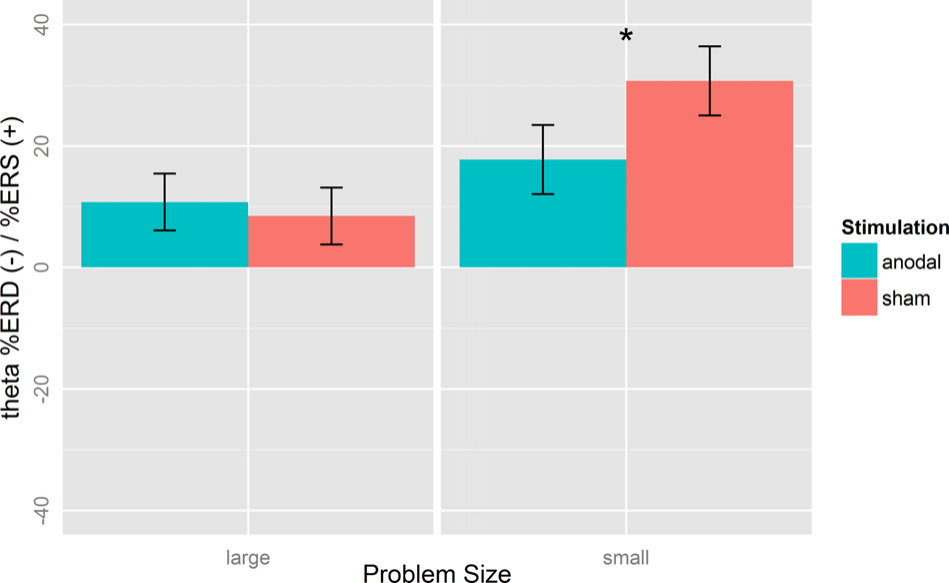
Mean %Event-related (de-)synchronization (%ERS/ERD) of the theta band (4–7 Hz) after anodal and sham stimulation for small and large problems at electrodes CP5/P5. Theta ERS in small problems was decreased after anodal compared to sham stimulation. Error bars indicate standard errors (SE). *p < 0.05, **p < 0.01.

Analysis of the stimulation effects revealed that in large problems, lower-alpha ERD was increased after anodal (M = -21.14, SD = 18.24) compared to sham stimulation (M = -6.86, SD = 20.98; *t*(22) = 3.43, *p* < 0.01, *d = 0*.*72*; Fig. 5 left panel). Theta ERS/ERD in large problems, in contrast, was unaffected by tDCS (*t*(22) = 0.49, *p* = 0.63, *d* = *0*.*10*; Fig. 6 left panel).

In small problems, theta ERS was reduced after anodal stimulation (M = 17.75, SD = 24.70) compared to sham stimulation (M = 30.72, SD = 23.98; *t*(22) = 2.27, *p* = 0.03, *d = 0*.*47*; Fig. 6 right panel). However, lower-alpha ERD in small problems was not affected by stimulation (*t*(22) = 1.06, *p* = 0.30, *d* = 0.22; Fig. 5 right panel).

To ensure that stimulation effects are due to changes in oscillatory EEG activity during task performance and not during rest, we performed the same analysis as above with power during the reference interval as the dependent measure. In this analysis, no effect reached significance. This suggests that the effects of stimulation are indeed related to task performance and not related to modulations in resting EEG power per se.

Taken together, the stimulation effects on task-related EEG activity dissociated between frequency bands depending on the problem size. TDCS increased lower-alpha ERD in large problems and decreased theta ERS in small problems.

## Discussion

The LPPC has been shown to constitute a key structure for arithmetic problem solving [18], with distinct activation patterns in the alpha and theta band while solving large and small problems [20–22]. In addition, anodal tDCS over this region has been successfully applied to improve performance in solving large arithmetic problems [58]. The aim of the present study was to combine and extend both research lines. Specifically, we investigated behavioral and electrophysiological effects of tDCS over the LPPC in arithmetic problems of small and large size, which can be assumed to be solved by retrieval or procedural strategies, respectively.

Our findings regarding the PSE are in line with previous studies. Solving large problems was clearly more error-prone and slower than solving small problems [10,11]. Moreover, large problems were accompanied by greater lower-alpha ERD, while small problems were characterized by greater theta ERS [20–22]. More important, however, are the findings regarding the effects of brain stimulation on large and small problems, which warrant a more detailed discussion.

### tDCS effects in large problems

Replicating the results of Hauser et al. [58], anodal stimulation of the LPPC compared to sham tDCS resulted in significant improvements in response latencies while solving large arithmetic problems. We extended these findings by demonstrating that these performance enhancements are accompanied by increases in lower-alpha ERD.

Generally, the finding of tDCS-induced changes in alpha activity is in line with previous research [74–78]. Based on the assumption that the amount of alpha ERD is a marker of cortical activation (see e.g. [34]), the present findings suggests that anodal tDCS during a complex arithmetic task increases cortical activation. With specific regard to findings relating lower-alpha ERD to basic attention and arousal [30–32,79], the observed effect might indicate modulations in basic attentional processes or resources. Considering another line of research showing that good cognitive performance is related to increased (lower) alpha ERD [32,79–81], it can be assumed that the increased lower-alpha ERD reflects elevated attention during complex problem solving, eventually resulting in improvements in response latency. This interpretation also corresponds to evidence showing improvements of attentional skills after anodal stimulation of the LPPC [82,83]. However, it must be noted that no effect was present for the solution rates. This might be due to the fact that response latencies might constitute a more sensitive measure for multi-step problems such as the large arithmetic problems used in the present study (i.e., problems in which several calculation steps are needed to arrive at the final solution). While solution rates mainly reflect correctness of the final solution, response latencies are more sensitive to the whole calculation process (e.g., number of steps, number of intermediate errors and speed of execution).

### tDCS effects in small problems

Anodal compared to sham stimulation resulted in significantly reduced solution rates in small problems while response latencies were unaffected. This finding contributes to the increasing evidence that anodal stimulation can not only have positive but—depending on task characteristics—also negative effects on cognitive performance [51–55]. For example, while several studies reported faster lexical retrieval and unaffected accuracy after anodal tDCS to the LPPC [84,85], Pisoni and Papagno [52] found retrieval impairments after the application of anodal tDCS to the left superior temporal gyrus. Specifically, the latter authors used a paradigm that required the subjects to overtly name a series of pictures presented either in semantically homogenous or in semantically mixed lists and found that anodal tDCS slowed down reaction times in semantically related lists. This was interpreted to reflect an increase of retrieval interference among semantically related stimuli. A similar mechanism may also account for the present findings. This interpretation is also supported by the concurring decrease in theta ERS we found in the present study. First, synchronous theta activity has been linked to various memory-related processes in general and to verbal and arithmetic fact retrieval in particular [20–22,25,86]. Second, EEG oscillations have been assumed to provide a mechanism for effective neural communication and information processing [87,88]. Consequently, it can be argued that decreased theta synchronization after anodal tDCS disturbs this information transfer by introducing more noise into the system [54], which then might lead to increased retrieval interference and probability to retrieve an erroneous answer to a particular arithmetic problem [6,7]. Although the specifics of this interpretation need to be examined, on the most basic level the data provide further support for the essential role of synchronous theta activity for (arithmetic) fact retrieval.

It must be noted that we found decreased solution rates after anodal tDCS, which is diverging from Pisoni et al.’s [52] finding of decreased response times. This difference might be related to the stimulus material used (verbal in the experiment by Pisoni et al. vs. numeric in the present study), the degree of training participants had with the material (trained in the experiment vs. overlearned arithmetic facts, respectively) or measurement time point (directly after stimulation vs. 15 minutes later, respectively). Furthermore, although significant, the solution rates were only reduced by about 1% after anodal compared so sham stimulation. We believe that this effect is worth reporting due to several reasons: First, it is line with recent evidence that cognitive enhancement in one cognitive function can occur at the expense of another one [51]. Second, the performance reduction in small problems after anodal compared to sham stimulation is very robust and consistent across individuals: It occurred in 15 out of 23 participants (in 3 participants, there were no performance changes, and in 5 participants, performance improved). Third, a modulation of performance in highly overlearned arithmetic problems can be expected to be rather small. Finally, from a statistical perspective, the effect is of medium size according to the suggestions by Cohen [89].

### Limitations

The first limitation of the present study is that we did not have an active control condition (i.e., vary the location of the active electrode), thereby introducing uncertainty about whether stimulation per se or stimulation of the LPPC specifically led to the observed effects. However, we are nonetheless confident that our findings arise predominantly due to stimulation of the LPPC due to several reasons: First, Hauser et al. [58] found that response latencies to solve large arithmetic problems were only decreased after anodal stimulation of the LPPC (as in the present study), while bilateral anodal or bilateral cathodal stimulation of the posterior parietal cortex did not affect arithmetic performance. Second, we found activation differences in the EEG at the stimulated areas in the frequency bands suggested by previous literature. Third, studies modeling the distribution of current flow by tDCS indicate that current densities—even though the overall induced electrical field is clearly non-focal—are largest under the active electrode [90]. Nonetheless, an active control condition should be included in future studies to further strengthen our conclusions.

Moreover, the relatively large, albeit conventional, size of the tDCS electrodes introduces uncertainty about how subregions of the LPPC, which have been differentially associated with the PSE (i.e., angular gyrus, intraparietal sulcus), were affected by the stimulation. For example, it could be that simultaneous activation of both areas resulted in interference effects. Future studies may use smaller electrodes to stimulate both areas more selectively to specifically test this hypothesis. Additionally, modelling of the current flow using realistic head models and the application of fMRI due to its advantageous spatial resolution could help to increase the spatial resolution of the stimulation. Furthermore, another concern is the relatively long stimulation duration (i.e., 30 min) used in the present study. It has been shown that the effects of tDCS do not always increase with longer stimulation durations, and in some cases might even reverse (e.g., decrease of excitability after anodal stimulation; see [91]). Thus, it is crucial to either use tested stimulation protocols or check the effects of DC stimulation with additional neurophysiological methods (e.g., EEG, fMRI).

Finally, in the present study, both blocks were analyzed for the behavioral data but only the first block was analyzed for the EEG data, because we expected different time-courses of the effects. On the one hand, since it is known that behavioral effects are rather long-lasting [92,93], we examined both blocks together to increase reliability. On the other hand, because it has been shown that tDCS effects on EEG oscillations diminish rather quickly and last only up to 15 minutes after stimulation [44], we intended to only investigate the first block. This, however, raises questions about the comparability between the behavioral and EEG data in the present study. Still, we believe that the evidence of previous studies relating theta synchronization to fact retrieval and alpha desynchronization to procedural calculation are robust enough to underline our interpretation. Nonetheless, to overcome this limitation, it will be crucial for future studies to decrease the time lag between stimulation and begin of EEG measurement, or to measure online- rather than after-effects of tDCS on arithmetic performance and EEG oscillations (see [35]).

## Conclusion

In sum, these findings demonstrate that the effects of brain stimulation are moderated by problem size and thus, provide further evidence that the LPPC is differentially involved in solving small and large arithmetic problems. These findings also add to the increasing evidence that the effects of brain stimulation on mathematical skills in general seem to strongly depend on the task characteristics and the involved neuro-cognitive processes [51]. This knowledge will be crucial in future applications of tDCS to support the remediation of mathematical learning disorders such as developmental dyscalculia [36].
